# Bariatric surgery prevents carotid wall thickness progression

**DOI:** 10.1007/s00508-022-02090-3

**Published:** 2022-10-07

**Authors:** Lukas Lunger, Andreas Melmer, Wolfgang Sturm, Claudia Lamina, Alexander Tschoner, Julia Engl, Armin Hönlinger, Clemens Engler, Peter Willeit, Stefan Kiechl, Johann Willeit, Dietmar Öfner, Heinz Wykypiel, Markus Laimer, Herbert Tilg, Christoph Ebenbichler

**Affiliations:** 1 Department of Internal Medicine I, Gastroenterology, Hepatology & Endocrinology, Medical University of Innsbruck, Anichstr. 35, 6020 Innsbruck, Austria; 2 Department of Urology, Technical University of Munich, Klinikum Rechts der Isar, Munich, Germany; 3 Institute of Genetic Epidemiology, Department of Genetics and Pharmacology, Medical University of Innsbruck, Innsbruck, Austria; 4 Department of Cardiac Surgery, Medical University of Innsbruck, Innsbruck, Austria; 5 Department of Neurology, Medical University of Innsbruck, Innsbruck, Austria; 6 Department of Visceral, Transplant and Thoracic Surgery, Centre of Operative Medicine, Medical University of Innsbruck, Innsbruck, Austria; 7 Department of Diabetes, Endocrinology, Nutritional Medicine and Metabolism, Inselspital, Bern University Hospital and University of Bern, Bern, Switzerland

**Keywords:** Carotid intima-media thickness, Bariatric surgery, Weight loss, Subclinical atherosclerosis, Carotid wall thickness progression

## Abstract

**Background:**

Bariatric surgery is a treatment option for patients with severe obesity and improves parameters of cardiovascular and/or metabolic disease. Carotid intima media thickness (C-IMT) is a surrogate measure of subclinical atherosclerosis. Previous studies showed short to mid-term arrest and even regression of C‑IMT progression following bariatric surgery. We aimed to investigate the long-term effect of weight loss on C‑IMT progression 10 years after bariatric surgery in comparison to a population-based control cohort.

**Methods:**

In total, 21 eligible patients were examined preoperatively, at 5 and 10 years after bariatric surgery. Anthropometric parameters, plasma triglycerides, total cholesterol, high-density lipoprotein cholesterol (HDL-C), insulin, and glucose were assessed at all three study visits. C‑IMT was measured via B‑mode scans of the common carotid artery. C‑IMT progression was measured in an age-matched and BMI-matched cohort selected from the population-based Bruneck study to compare with changes in C‑IMT progression after bariatric surgery.

**Results:**

C‑IMT remained stable over the 10-year observation period after bariatric surgery. The control cohort showed a significant C‑IMT progression over 10 years. The difference in C‑IMT progression over 10 years was significant (*p* < 0.01) between both cohorts.

**Conclusion:**

Weight loss induced by bariatric surgery halts the natural progression of C‑IMT over a 10-year observation period.

**Supplementary Information:**

The online version of this article (10.1007/s00508-022-02090-3) contains supplementary material, which is available to authorized users.

## Introduction

Overweight and obesity account for 80% of cases of type 2 diabetes (T2DM), 35% of ischemic heart disease and 55% of hypertension worldwide [1–3]. Sustained weight loss induced by bariatric surgery is an effective treatment option for obese patients. Bariatric surgery was able to significantly reduce cardiovascular mortality and morbidity, especially due to reductions of the incidence of diabetes, ischemic heart disease and cancer [4–8].

Carotid intima media thickness (C-IMT), as determined by ultrasound, is a well-established surrogate measure of subclinical atherosclerosis and associated with an almost twofold increased risk for cardiovascular disease in men with a C-IMT ≥ 1 mm as compared with a C-IMT < 1 mm (odds ratio, OR 1.85; confidence interval, CI: 1.28–2.69) [9–12].

Large trials and meta-analyses of individual patients’ data revealed a significant association between changes in C‑IMT and cardiovascular risk in the general population. Moreover, it was calculated that the natural progression of C‑IMT approximates to 0.08–0.1 mm per 10 years in both women and men [12–14].

Evidence furthermore illustrated higher C‑IMT values in obese patients as well as significant decreases in C‑IMT following bariatric surgery [15, 16]. Data from a meta-analysis reported reductions of C‑IMT by 0.03 mm following both surgically and conservatively induced weight loss over a 20-month observation period [17]. A recent prospective study reported a decrease in C‑IMT over a 12-month observation period in women undergoing Roux-en‑Y gastric bypass (RYGB) and sleeve gastrectomy, as compared with conservative weight loss [18]. However, generalizability of these results is limited especially by short observation periods (< 18 months) following bariatric surgery and the absence of control cohorts [16, 19].

The present prospective study addressed these limitations by observing C‑IMT-progression over a 10-year period in 2 cohorts: patients undergoing bariatric surgery and a matched control cohort selected from a population-based study.

## Methods

### Patients

A previous study conducted at our clinic evaluated C‑IMT and other cardiometabolic parameters 5 years following bariatric surgery [19]. For this current analysis, all patients participating in the 5‑year evaluation were contacted. Moreover, three patients undergoing bariatric surgery in the same time period and not included in the 5‑year evaluation were asked to participate in this study.

Inclusion criteria were a body mass index (BMI) > 35 kg/m^2^ and at least one obesity-associated comorbidity or a BMI > 40 kg/m^2^. Exclusion criteria were overt diabetes prior to surgery, uncontrolled hypertension (> 160/90 mm Hg), history of cardiovascular disease, secondary causes of obesity, pregnancy, elevated liver enzyme activities (> 3 times upper limit of normal), lipid lowering or antipsychotic medication, acute or chronic liver disease, and a history of an average alcohol consumption of more than 20 g alcohol per day. Patients with acute infectious and inflammatory diseases were excluded by taking a medical history and performing physical and laboratory examinations. All baseline procedures were performed on the same day within a 2-month period prior to surgery.

Bariatric surgery was performed between 1998 and 2004 at the University Clinics for Visceral, Transplant, and Thoracic Surgery at the Medical University of Innsbruck.

Written informed consent was obtained from each participant prior to any study-related activities. All procedures performed in the present study involving human participants were in accordance with the ethical standards of the institutional and/or national research committee and with the 1964 Helsinki declaration and its later amendments and were performed after written approval of the ethics committee at the Medical University of Innsbruck.

### Control cohort

Participants from the population-based Bruneck study served as a control cohort. The Bruneck study is a prospective, population-based study to investigate the epidemiology and pathogenesis of atherosclerosis and related traits [20, 21]. The study was initiated in 1990 in an age-matched and sex-stratified random sample of 1000 subjects recruited from the entire population of Bruneck, a small town in southern Tyrol (Bolzano Province, Italy). Follow-up examinations were performed every 5 years. In selected cases, C‑IMT measurements were available from the years 1995, 2000, and 2005. To increase comparability to bariatric patients in the present study, 12 female and 9 male participants were chosen and matched by age and BMI (e.g. the female participant with the highest BMI in the Bruneck study was matched to the female participant with the highest BMI in the patient group). Due to the lack of participants with severe obesity in the Bruneck study, the BMI range of the control cohort was matched with the BMI range at 5–10 years after bariatric surgery. Therefore, the statistical model was adjusted to account for these significant differences at baseline (preoperative BMI 40.4 ± 6.1 kg/m^2^ vs. 32.8 ± 2.4 kg/m^2^ in the control cohort). Similarly, age ranges differed between both cohorts (age range at baseline in bariatric patients: 21–56 years vs. 40–79 years in the control cohort). As exact matching by age was not feasible, the conditional three-level growth model was additionally adjusted for this incomplete overlap (see section on statistical methods for details).

### Anthropometric measures and demographic information

In both bariatric surgery patients and controls, anthropometric parameters included body weight, measured to the nearest 0.1 kg using an electronic scale and body height, measured to the nearest 0.1 cm using a tape line. The BMI was calculated dividing body weight by body height in meters squared. All bariatric surgery patients were instructed to notify the study center of changes in routine medication use, especially regarding the intake of lipid lowering or blood pressure altering medications.

### Laboratory analyses

In bariatric surgery patients, blood samples were drawn in the morning in the fasting state from the antecubital vein into EDTA and serum tubes (1.6 mg/ml) and centrifuged immediately after collection at 3000 rpm for 10 min at 4 °C. Plasma samples were stored at −80 °C until being used for assaying.

Serum and plasma parameters were measured according to standards as defined by the Central Laboratory Unit of the Medical University of Innsbruck. Plasma triglycerides, total cholesterol, and high-density lipoprotein cholesterol (HDL‑C) were quantified using a commercially available enzymatic kit (Roche Diagnostic Systems, Basel, Switzerland). Low-density lipoprotein cholesterol (LDL‑C) was calculated using the Friedewald formula. Plasma glucose was measured by the hexokinase method on a Cobas MIRA analyzer (Roche Diagnostic Systems, Basel, Switzerland).

### Carotid intima media thickness

The C‑IMT was measured as described elsewhere [22]. Longitudinal B‑mode scans of the common carotid artery were obtained using a standard Acuson Sequoia 512 system (Acuson, Mountain View, CA, USA) and a 9.0-MHz, linear-array transducer. C‑IMT was defined as the distance between the junction of the lumen and the intima and that of the media and adventitia. Maximum C‑IMT at the left and right carotid artery was measured just proximal to the carotid bulb (the last 2 cm) and then averaged to obtain the mean maximum C‑IMT. The same procedure was applied in the Bruneck study.

### Statistical methods

Deviations from normality for C‑IMT measurements were tested by using the Shapiro-Wilks test within cases and controls separately. Variables are given as mean ± standard deviation. Student’s t‑test for paired observations was performed to compare mean C‑IMT at baseline with respective C‑IMT measurements at the 5‑year and 10-year follow-up visits in both case and control groups separately. To determine a different effect of time on C‑IMT progression between cases and controls, an interaction term between time and case-control group status on C‑IMT change was introduced in a conditional three-level growth model, accounting for repeated measures within each person and for 1:1 matching design (package nlme in R, R Core Team <R-core@R-project.org>). This model was additionally adjusted for age at baseline and BMI at each of the follow-up time points, since matching could not be perfectly done on these factors.

To address the potential of selection bias in the patients recruited for the 10-year follow-up as well as to highlight differences between cases and controls, quantitative parameters were compared using the Wilcoxon test, qualitative parameters with Fisherʼs exact test. All analyses were performed using IBM SPSS 24 (IBM, Armonk, NY, USA) or R Version 3.0.1 (R-Development Core Team, R Foundation, Vienna, Austria). Illustrations were designed using GraphPad Prism 7.00 (GraphPad Software, Inc. La Jolla, CA, USA) and R.

## Results

### Anthropometric and metabolic measures

Of the *n* = 52 patients participating in the 5‑year follow-up [19], 18 patients agreed to participate in this current 10-year evaluation. Moreover, 3 patients with a 5-year follow-up following bariatric surgery (independent of the previously published work) agreed to participate in this study. A total of 12 female and 9 male subjects undergoing bariatric surgery with a mean age of 36.4 ± 9.5 years were then finally included in this prospective 10-year analysis. Clinical and metabolic parameters assessed before, 5 years and 10 years after bariatric surgery are presented in Table 1. To address the possibility of selection bias, a comparison of the *n* = 18 patients agreeing to participate with the *n* = 34 patients lost to follow-up is presented in supplementary Table 1. Exploratory statistical testing revealed an equal distribution of all investigated parameters including current weight (and thereby weight loss) as well as cardiometabolic risk markers distributed across both groups, underlining the random aspect of patient selection in this study.

**Table 1:** Descriptive statistics (mean ± SD and [25, 50, 75] percentiles for quantitative parameters, *n* (%) for sex)

		*n* cases/controls	Patient group/cases	Subgroup Bruneck study/controls^b^	*p*-value for difference^a^
Age at baseline [years]		21/21	36.4 ± 9.5 [29, 37, 42]	50.4 ± 3.9 [49, 51, 53]	< 0.001
Sex (female)		21/21	12 (57.1%)	12 (57.1%)	1
BMI [kg/m^2^]	Baseline	21/21	40.4 ± 6.1 [38.31, 40.86, 43.10]	32.8 ± 2.4 [31.2, 31.8, 33.9]	< 0.001
	5 years	19/21	32.0 ± 4.1 [28.62, 31.99, 34.19]	32.2 ± 2.3 [30.3, 31.9, 33.7]	0.8
	10 years	21/21	33.3 ± 5.0 [29.4, 33.4, 36.9]	33.3 ± 3.0 [31.1, 33.5, 35.5]	0.9
Glucose [mg/dl]	Baseline	21/21	98.1 ± 11.0 [93, 94, 103]	106.8 ± 15.3 [98, 101, 113]	0.049
	5 years	19/21	98.5 ± 24.0 [88.5, 93.0, 99.5]	102.5 ± 12.0 [94, 99, 108]	0.03
	10 years	21/21	97.6 ± 25.6 [82, 91, 102]	105.8 ± 14.5 [96, 101, 109]	0.01
TC [mg/dl]	Baseline	21/21	193.5 ± 40.3 [164, 198, 228]	215.8 ± 42.0 [185, 212, 233]	0.1
	5 years	19/21	177.5 ± 28.1 [163.5, 180, 198]	223.7 ± 30.5 [208, 221, 246]	< 0.001
	10 years	21/21	192.4 ± 26.9 [173, 192, 210]	214.6 ± 33.9 [194, 213, 226]	0.03
HDL‑C [mg/dl]	Baseline	21/21	49.5 ± 12.2 [42, 49, 58]	47.7 ± 14.1 [37, 47, 51]	0.3
	5 years	19/21	47.2 ± 12.7 [38, 44, 55]	50.1 ± 13.1 [42, 47, 59]	0.6
	10 years	21/21	59.2 ± 15.8 [49, 60, 73]	57.0 ± 15.1 [45, 57, 60]	0.4
LDL‑C [mg/dl]	Baseline	21/20	118.2 ± 34.4 [91, 118, 146]	138.5 ± 36.5 [105.6, 132.6, 165.5]	0.1
	5 years	19/21	109.1 ± 25.0 [91.5, 110.0, 124.5]	142.2 ± 28.7 [116, 138, 151]	0.001
	10 years	21/21	120.9 ± 25.8 [101, 119, 146]	128.8 ± 33.6 [108, 121, 136]	0.7
TG [mg/dl]	Baseline	21/21	124.4 ± 52.3 [87, 120, 153]	168.1 ± 128.4 [95, 120, 157]	0.6
	5 years	19/21	107.5 ± 48.1 [76.5, 99.0, 124.5]	154.1 ± 70.0 [93, 148, 193]	0.03
	10 years	21/21	93.4 ± 44.0 [57, 87, 116]	141.5 ± 66.7 [98, 141, 167]	0.004

*BMI* body mass index, *HDL‑C* high density lipoprotein-cholesterol, *LDL‑C* low density lipoprotein-cholesterol, *TC* total cholesterol, *TG* triglycerides

^a^Quantitative parameters were compared using Wilcoxon test, Qualitative parameters with Fisherʼs exact test

^b^All values were obtained from subjects in a fasting state

Nineteen patients received adjustable gastric banding (AGB) (90.5%), whereas Roux‑Y gastric bypass surgery (RYGB) was performed in 2 patients (9.5%).

Mean BMI in the case group decreased from baseline to the 5‑year follow-up per patient by −9.3 ± 6.5. kg/m^2^ The mean BMI decrease per patient from baseline to the 10-year follow-up was −7.1 ± 7.7 kg/m^2^ indicating a slight increase BMI increase after the 5‑year follow-up. Baseline, 5‑year and 10-year follow-up clinical and metabolic parameters of the control group (Bruneck study) are reported in Table 1. The control group was older than the case group at study start (50.4 ± 3.9 years) and consisted of 12 female and 9 male participants. Mean BMI in the control group was lower at study start (32.8 ± 2.4 kg/m^2^) compared to the case group (40.4 ± 6.1 kg/m^2^; *p* < 0.001). The BMI of the control group remained stable over time. The mean BMI change in the control group between study start and the 5‑year follow-up was −0.7 ± 2.0 kg/m^2^ and between start and 10-year follow-up + 0.5 ± 1.8 kg/m^2^. Mean BMI of the control group was comparable to the case group at the 5‑year (32.2 ± 2.3 kg/m^2^) and 10-year follow-up (33.3 ± 3.0 kg/m^2^; see Table 1). BMI at the 10-year follow-up visit was significantly lower in women compared to men (4.6 ± 2.0 kg/m^2^ standard error difference, *p* = 0.03).

Overall, 1 of 21 patients reported intake of a lipid lowering agent at the 5‑year follow-up visit.

### C-IMT during the observation period

**Fig. 1:**
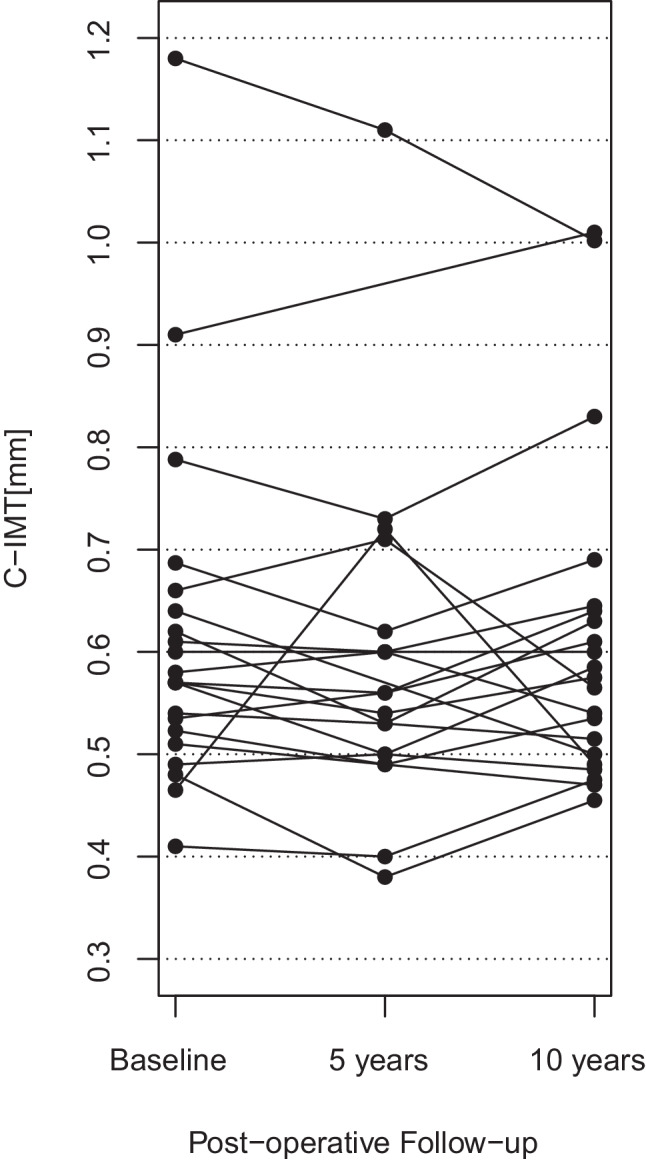
Individual C‑IMT changes after bariatric surgery during the 10-year observation period. *C‑IMT* carotid intima media thickness

**Fig. 2:**
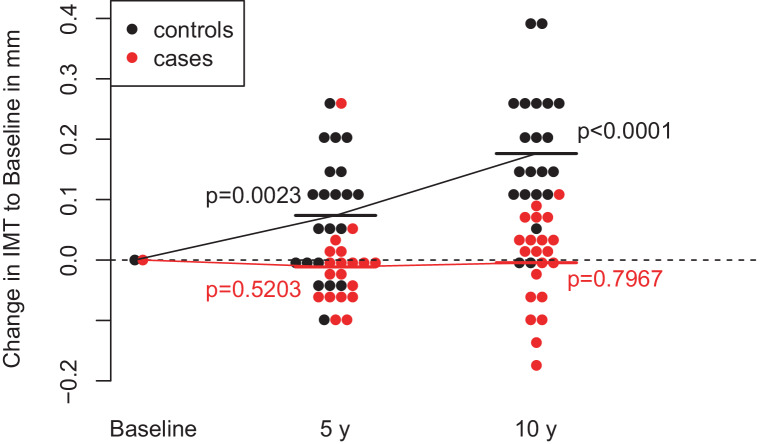
Change in C‑IMT in mm to Baseline after 5 years and 10 years of follow-up in cases and controls; mean values are shown for cases and control group as horizontal lines; *p*-values are derived from paired t‑tests comparing the respective follow-up-values with baseline values. *C‑IMT* carotid intima media thickness

Results of C‑IMT measurements are listed in Table 2. Individual changes of C‑IMT are depicted in Fig. 1. Changes in C‑IMT after bariatric surgery and in the control cohort are illustrated in Fig. 2. C‑IMT remained stable from baseline to 5 years (*p*-value of paired t‑test: 0.52) and 10 years (*p*-value of paired t‑test: 0.79) after bariatric surgery. In the control cohort, C‑IMT increased significantly from baseline to 5 years (*p* < 0.01) and 10 years (*p* < 0.001).

**Table 2:** C‑IMT across the 10-year observation period (mean ± SD and [25, 50, 75] percentiles)

C‑IMT [mm]	n	Cases	Mean C‑IMT difference for cases (*p*-value)	Controls	Mean C‑IMT difference for controls (*p*-value)
At baseline	21	0.62 ± 0.17 [0.52, 0.57, 0.64]	–	0.84 ± 0.11 [0.8, 0.8, 0.9]	–
At 5 years	19/21^a^	0.59 ± 0.16 [0.50, 0.56, 0.61]	−0.01 ± 0.08 (0.52)	0.91 ± 0.14 [0.8, 0.9, 1.0]	0.07 ± 0.10 (< 0.01)
At 10 years	21	0.61 ± 0.16 [0.50, 0.57, 0.64]	−0.00 ± 0.08 (0.79)	1.01 ± 0.18 [0.95, 0.95, 1.05]	0.18 ± 0.11 (< 0.001)

*C‑IMT* carotid intima media thickness

^a^*n* = 19 in cases, *n* = 21 in controls

According to a conditional three-level growth model, change in C‑IMT differed significantly between patients undergoing bariatric surgery and the control cohort (*p* < 0.01) with an estimated increase of C‑IMT by 0.0196 mm per year for controls and 0.0003 for cases (effects are age-adjusted and BMI-adjusted).

## Discussion

The C‑IMT remained stable over 10 years after bariatric surgery, while a significant natural increase in C‑IMT was observed in a population-based control cohort.

These results extend current knowledge about the effect of bariatric surgery on C‑IMT and continues results of our own previously published study. Habib et al. confirmed the rapid regression of C‑IMT during the early 6‑month postoperative period. This regression was then sustained across until the end of the 24-month observation period, suggesting a long-term and sustained risk-reducing effect of bariatric surgery [23]. The comprehensive meta-analysis of surgical and non-surgical weight loss interventions by Skilton et al. with a mean follow-up of 20 months and an average weight loss of 16 kg (95% CI 9.4–22.5 kg) reported a reduction of C‑IMT by 0.03 mm following weight loss. The results were similar between both conservative and surgical interventions [17]. Furthermore, the systematic review by Lupoli et al. reported an association between surgically induced weight loss and improvement of subclinical atherosclerosis markers [16]. In a previous study we were able to show that C‑IMT progression is slowed and, in some individuals, even halted 5 years after bariatric surgery [19].

These effects may in part be mediated by improvements of obesity-associated cardiovascular risk factors, which may subside in the long term [24–26]. For instance, bariatric surgery has been shown to reduce and, in some cases, resolve obesity-associated comorbidities such as diabetes mellitus, but also hypertension, dyslipidemia or obstructive sleep apnea syndrome [5]. In previous studies it was observed that short-term modifications of C‑IMT are particularly associated with changes in anthropometric parameters (weight, BMI, waist circumference, systolic and diastolic blood pressure) [16]. Also, changes in C‑IMT were shown to be associated with changes in lipoprotein profiles [27]. Recent findings suggest that particularly HDL-associated factors may help to explain the beneficial effects of surgical weight loss on C‑IMT. Metabolic surgery significantly improves both the quantity as well as the function of HDL subspecies [28].

A recently published observational study investigated the long-term impact of statin treatment on C‑IMT in a large cohort of men aged > 70 years. The results of this study showed that statins can prevent the progression of C‑IMT over a period of 10 years as compared to non-statin treated controls [29]. To avoid this bias, intake of lipid lowering drugs was an exclusion criterion before participating in this study. At the 5‑year follow-up, only 1 of 21 patients reported intake of lipid lowering agents. Of note, in this study, C‑IMT remained stable over time, despite no intake of lipid lowering drugs following bariatric surgery.

A limiting factor of this study may be the small sample size in the intervention group as well as the imperfect matching of the Bruneck study to the patient cohort due to the incomplete age range (14 years difference between intervention and control group) and BMI overlap between both cohorts. Available data suggest that age might be a major contributing factor of C‑IMT. In this study, the mean age in the intervention group was significantly lower as compared to the control group. This might have contributed to the observed difference in C‑IMT between the intervention and the control group. However, as age was not correlated to the nonvarying stable parameter C‑IMT over time in the intervention group, we assume a dominant impact of weight loss and metabolic parameters on C‑IMT progression. Progression of C‑IMT appears to occur proportionally to increased age; however, these observations stem from cross-sectional studies only and might therefore not be comparable to these current results. It is therefore possible, although unlikely, that there might be some bias due to the increased cardiovascular risk factors observed in the older control group. As highlighted in a paper by Kozakova et al. a change in C‑IMT might be more relevant among women throughout the sixth decade of life [30].

Similarly, a higher BMI might accelerate C‑IMT progression. The reported significant difference in BMI at baseline poses a particular limitation, especially due to the unavailability of untreated controls with comparable severe obesity. However, we additionally adjusted the model for BMI and age at baseline to account for the abovementioned differences. Despite the lack of participants with severe obesity in the Bruneck study to match the observed baseline intervention group BMI, the resultant BMI range was adequately matched at the 5‑year and 10-year follow-up. Using this optimized best-matching approach, we were therefore still able to compare the progression of C‑IMT after bariatric surgery to the natural progression without an intervention over the same time span. Another limitation may be the relatively elevated cardiovascular risk factors in the control group as compared to the intervention group. This might be explained by the fact that patients undergoing bariatric surgery are subjected to stringent dietary restrictions both in the preoperative and postoperative stage, whereas the Bruneck study reflects real-world, population based and non-intervention data. Also, the measurement of cholesterol efflux and HDL subspecies may have substantially strengthened the discussion.

An additional limiting factor might be the prevalent type of surgery in this study: The AGB has been shown to yield less weight loss as compared to RYGB in the long term, which could significantly impact C‑IMT dynamics [5]. However, in this study, even though > 90% of patients had undergone AGB, improvements in cardiovascular and metabolic parameters remained significant, accompanied by a halt of C‑IMT progression.

Major strengths of this study were the comparison of C‑IMT measurements obtained across 3 time points with an adequately matched control group as well as the substantial observation period of 10 years. This is of particular importance, considering that C‑IMT as a parameter of atherosclerotic vessel wall remodeling is a chronic, rather than a short-term process.

## Conclusion

In conclusion, bariatric surgery was capable to inhibit C‑IMT progression over a 10-year observation period compared to a natural C‑IMT increase in a population-based control cohort.

## Supplementary Information

Supplementary table 1: Comparison of *n* = 18 patients agreeing to the 10-year follow-up visit with the *n* = 34 patients not available for the intended 10-year follow-up. Quantitative parameters are given as median (IQR), qualitative parameters (sex, non-smoking status) are given as *n* (%)
