# Supplementary material for: Bariatric surgery prevents carotid wall thickness progression
Source: Wien Klin Wochenschr. 2022 Oct 7;135(5-6):151–7. doi: 10.1007/s00508-022-02090-3 (PMC10020246; doi:10.1007/s00508-022-02090-3)
Supplement: Supplementary file 1 — Supplementary table 1: Comparison of n = 18 patients agreeing to the 10-year follow-up visit with the n = 34 patients not available for the intended 10-year follow-up. Quantitative parameters are given as median (IQR), qualitative parameters (sex, non-smoking status) are given as n (%) [file 508_2022_2090_MOESM1_ESM.docx]

| Supplementary table 1: Comparison of n=18 patients agreeing to the 10-year follow-up visit with the n=34 patients not available for the intended 10-year follow-up. Quantitative parameters are given as median (IQR), qualitative parameters (sex, non-smoking status) are given as n (%). | | | | |
| --- | --- | --- | --- | --- |
|  | Total | Patients (n=34) lost to follow-up/NA for the 10-year follow-up | Patients (n=18) agreeing to the 10-year follow-up | p-value for difference* |
| Age at baseline [years] | 34 vs 18 | 34.0 (13) | 35.0 (14) | 0.8 |
| Sex (female) | 34 vs 18 | 29 (85) | 11 (61) | 0.049 |
| Non- smoking status at baseline | 34 vs 18 | 23 (68) | 11 (61) | 0.4 |
| BMI at five years PO [kg/m²] | 34 vs 18 | 31.8 (9.7) | 31.9 (5.9) | 0.9 |
| Glucose at five years PO [mg/dl] | 34 vs 18 | 89.0 (14) |  |  |
|  |  |  | 91.5 (12) | 0.2 |
|  |  |  |  |  |
| TC at five years PO [mg/dl] | 34 vs 18 | 174.5 (37) | 186 (38) | 0.7 |
| HDL-C at five years PO [mg/dl] | 34 vs 18 | 53.0 (17) | 44.5 (21) | 0.2 |
| LDL-C at five years PO [mg/dl] | 34 vs 18 | 100 (54) | 112 (39) | 0.8 |
| TG at five years PO [mg/dl] | 34 vs 18 | 91.5 (60) | 94 (50) | 0.6 |
| IMT at five years PO [mm] | 34 vs 18 | 0.55 (0.16) | 0.53 (0.11) | 0.5 |
| Systolic pressure at 5 years PO [mmHG] | 34 vs 18 | 130.0 (22) | 124 (16) | 0.1 |
| Diastolic pressure at 5 years PO [mmHG] | 34 vs 18 | 80 (17) | 80 (16) | 0.3 |
| * Quantitative parameters were compared using Wilcoxon test, Qualitative parameters with Fishers exact test  Abbreviations: BMI = Body mass index. HDL-C = high density lipoprotein – cholesterol. LDL-C = low density lipoprotein – cholesterol. TC = Total cholesterol. TG = Triglycerides.  **All values were obtained in a fasting state. | | | | |
